# Leucine zipper and ICAT domain containing (LZIC) protein regulates cell cycle transitions in response to ionizing radiation

**DOI:** 10.1080/15384101.2019.1601476

**Published:** 2019-04-19

**Authors:** George Skalka, Holly Hall, Joanna Somers, Martin Bushell, Anne Willis, Michal Malewicz

**Affiliations:** a MRC Toxicology Unit, University of Cambridge, Leicester, UK; b Beatson Institute for Cancer Research, Glasgow, UK

**Keywords:** Ionising radiation, DNA damage, cell cycle, checkpoint, G2/M, LZIC

## Abstract

Common hallmarks of cancer include the dysregulation of cell cycle progression and the acquisition of genome instability. In tumors, G1 cell cycle checkpoint induction is often lost. This increases the reliance on a functional G2/M checkpoint to prevent progression through mitosis with damaged DNA, avoiding the introduction of potentially aberrant genetic alterations. Treatment of tumors with ionizing radiation (IR) utilizes this dependence on the G2/M checkpoint. Therefore, identification of factors which regulate this process could yield important biomarkers for refining this widely used cancer therapy. Leucine zipper and ICAT domain containing (LZIC) downregulation has been associated with the development of IR-induced tumors. However, despite LZIC being highly conserved, it has no known molecular function. We demonstrate that LZIC knockout (KO) cell lines show a dysregulated G2/M cell cycle checkpoint following IR treatment. In addition, we show that LZIC deficient cells competently activate the G1 and early G2/M checkpoint but fail to maintain the late G2/M checkpoint after IR exposure. Specifically, this defect was found to occur downstream of PIKK signaling. The LZIC KO cells demonstrated severe aneuploidy indicative of genomic instability. In addition, analysis of data from cancer patient databases uncovered a strong correlation between LZIC expression and poor prognosis in several cancers. Our findings suggest that LZIC is functionally involved in cellular response to IR, and its expression level could serve as a biomarker for patient stratification in clinical cancer practice.

## Introduction

DNA damage can be induced by numerous internal and external sources, such as the collapse of DNA replication forks and exposure to exogenous high-energy radiation [1]. Upon recognition of DNA damage, cells mount a coordinated response of adaptive signaling pathways collectively termed the DNA damage response (DDR) [2]. In addition to DNA break repair pathways, the DDR includes a series of specialized DNA damage sensing and signaling proteins which arrest the cell at specific checkpoints during the cell cycle [3]. These checkpoints allow for the completion of DNA repair prior to DNA replication and cell division [4]. Importantly, checkpoints will activate depending on the specific modalities of damage, for example, activation of the G2/Mitosis (G2/M) checkpoint is associated with the exposure of cells to high-energy radiation [5,6]. The break-down of cell cycle checkpoint control can be a precursor to multiple pathological conditions, such as tumorigenesis. Most widely studied is the loss of p53 and p21 proteins resulting in failure to activate G1 checkpoint [7,8]. In these situations, the G2/M checkpoint becomes critically important for the maintenance of cell genome stability [9].

Activation and maintenance of the G2/M checkpoint is controlled by protein kinases. The phosphatidylinositol 3-kinase-related kinase (PIKK) family is activated following identification of DNA damage. Ataxia-telangiectasia mutated (ATM) and Ataxia-telangiectasia mutated and Rad3 related (ATR) are members of this family. One function of these proteins following damage is to activate the G2/M checkpoint signaling cascade [10]. To maintain the signal transduction cascade the master regulator of the G2/M signaling cascade, checkpoint protein 1 kinase (Chk1), is activated [11]. This requires phosphorylation of two serine residues at positions 345 (S345) and 317 (S317), which is mediated by ATR and ATM. Importantly, phosphorylated Chk1 is essential for the activation of the G2/M checkpoint in response to treatment with ionizing radiation (IR) [12]. Chk1 functions by phosphorylating specific inhibitory sites within cell cycle control proteins. An example of this is the phosphorylation of WEE1 by Chk1 in response to damage, which in turn induces an inhibitory phosphorylation event on Tyrosine 15 (Tyr15) of CDC2, inhibiting entry into mitosis [13]. The G2/M checkpoint is maintained until DNA repair has been completed at which point the checkpoint is deactivated and cells resume normal cell cycle. Release from cell cycle arrest is conducted by various protein phosphatase family members, such as PP2 and PP1. This activity is through the removal of phosphorylation from inhibitory sites on cell cycle controllers [14,15]. Incorrect functioning of any step within this process can lead to a dysfunctional G2/M checkpoint, which can result in chromosomal abnormalities, e.g., aneuploidy [16]. Cellular reaction to IR encompasses both direct repair response and induction of checkpoint signaling cascade. While many proteins which mediate these responses have been identified, further investigation into these response pathways is required to understand the nuances of control.

One protein, which was linked to cellular IR response, is the Leucine zipper and ICAT domain containing (LZIC) protein [17]. LZIC is a putative member of the WNT signaling family [17]. The LZIC protein is composed of 190 amino acids (21 kDa) and contains two domains, an N-terminal coiled-coil and a C-terminal ICAT-like domain (Supplemental Figure 1A). Unlike ICAT protein, which antagonizes WNT signaling by binding and inhibiting ß-catenin, LZIC protein does not interact with ß-catenin [18]. Furthermore, in a rat model of IR-induced osteosarcoma reduced LZIC expression was associated with the onset of oncogenesis [19–21]. To investigate the function of LZIC protein we have employed CRISPR technology to derive LZIC knock-out (KO) cell lines. Our data show that LZIC is a component of the cellular response to IR. LZIC deficient cells show dysregulated transcription after IR treatment and fail to efficiently maintain the G2/M checkpoint, with the generation of severe genomic instability. Finally, analysis of patient databases identified a positive correlation between LZIC expression and average patient survival time in a number of cancers, suggesting that LZIC expression could serve as a biomarker for patient stratification.

## Results

### LZIC deletion leads to gene expression changes following treatment with ionizing radiation

LZIC is a putative member of the WNT signaling pathway, which typically regulates the activity of TCF/LEF family transcription factors and has been implicated in response to IR [22]. As such, we sought to determine the impact of LZIC loss on late transcriptional regulation following IR [23]. To address this question, CRISPR was used to generate an HEK293 cell line with a deletion of LZIC (LZIC KO Clone 1) and a control line, which has undergone the CRISPR process, but with no LZIC deletion (Supplementary Figure 1B).

**Figure 1.**
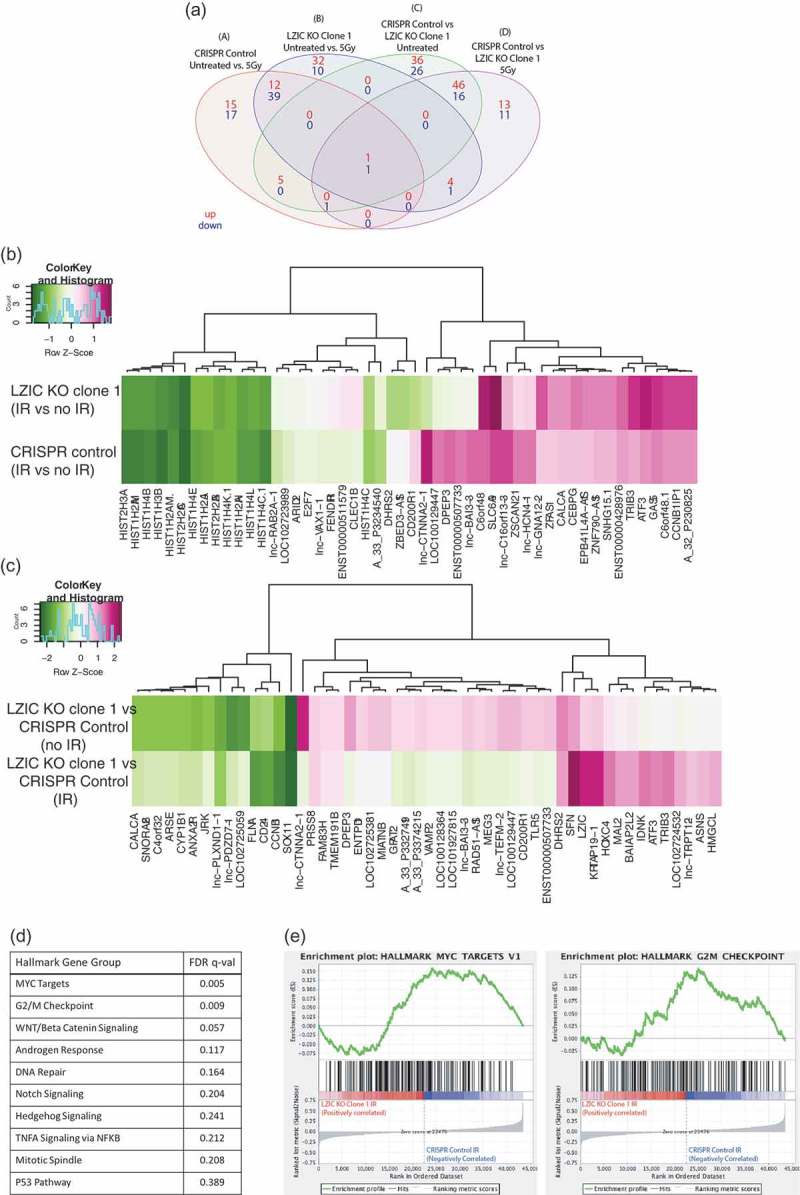
The loss of LZIC expression alters transcriptome under basal conditions and in response to ionizing radiation. (A) Venn diagram comparing the numbers of genes with significantly altered log-fold changes between LZIC KO clone vs CRISPR control cells in control and IR conditions. (B) Heatmaps representing Z-scores for genes comparing transcriptomic profiles in CRISPR control to LZIC KO Clone 1 cell lines in response to 5 Gy IR. (C) Heatmaps representing Z-scores for comparison of transcriptional profiles between CRISPR control and LZIC KO Clone 1 either under basal condition or following IR exposure. (D) Hallmark gene groups analyzed in GSEA and their associated FDR q-value (E) Barcode plots for significant GSEA hallmark gene groups. Microarray was repeated on two separate biological repeats with two technical repeats of each condition.

Differential expression was determined by comparing whole genome expression profiling 24 h following 5 Gy of IR with cells which were left untreated for both LZIC KO and the CRISPR control (Figure 1A). Genes involved in the response to DNA damage were found to be differentially regulated following treatment with IR in the CRISPR control (Figure1, Group A). Following the loss of LZIC expression, we detected 42 genes which are uniquely regulated (Figure 1, Group B). To further investigate the relationship between these groups a z-score analysis was conducted. This indicates strongly related clusters of genes between the cell lines, with the reduced expression of histone subunits being most conserved following treatment with IR (Figure 1B). However, differences between the two cell lines can be observed, with a dysregulation of several long-non-coding (lnc) RNAs and a downregulation DHRS2, which is involved in the p53 regulatory cascade, in LZIC KO conditions.

To directly investigate the loss of LZIC on the transcriptome, the differential expression between LZIC KO cells and CRISPR control was determined. In untreated conditions, we identified a total of 62 unique genes which are differentially regulated following LZIC loss (Figure 1A, Group C). Genes involved in neuronal development, such as FOXQ1 and Peripherin, are present in line with previous reports of LZIC function. In comparison, we found 24 unique genes which are differentially regulated in response to IR following LZIC loss (Figure 1A, Group D). This group includes genes such as PLK2, which has a role in cell division. Among the genes identified following LZIC KO, regardless of treatment with IR, were SFN and CCBN1, which are critical regulators of the G2/M checkpoint (Figure 1C). The 10 most significantly altered transcripts from each unique group are highlighted in table form (Supplementary Figure 1C). Differential expression of 10 mRNAs was validated by qPCR (Supplementary Figure 2A & 2B).

To examine specific pathways which were dysregulated in LZIC KO we utilized GSEA (Gene set enrichment analysis) using MSigDB (Molecular Signatures Database) hallmark gene sets. This revealed that LZIC KO causes alteration of MYC signaling and G2/M checkpoint pathways following treatment with IR (Figure 1D & 1E) [24,25]. This analysis was also performed on the basal conditions, identifying MYC targets and E2F targets, suggesting that MYC alterations are LZIC dependent, with changes to G2/M and E2F targets being treatment dependent (Supplementary Figure 2C).

Overall our transcriptome analysis found that LZIC KO cells had an altered transcriptional profile under both basal conditions and after treatment with IR, with a particular focus on cell cycle regulation.

### LZIC loss leads to increased release from G2/M phase in response to IR

**Figure 2.**
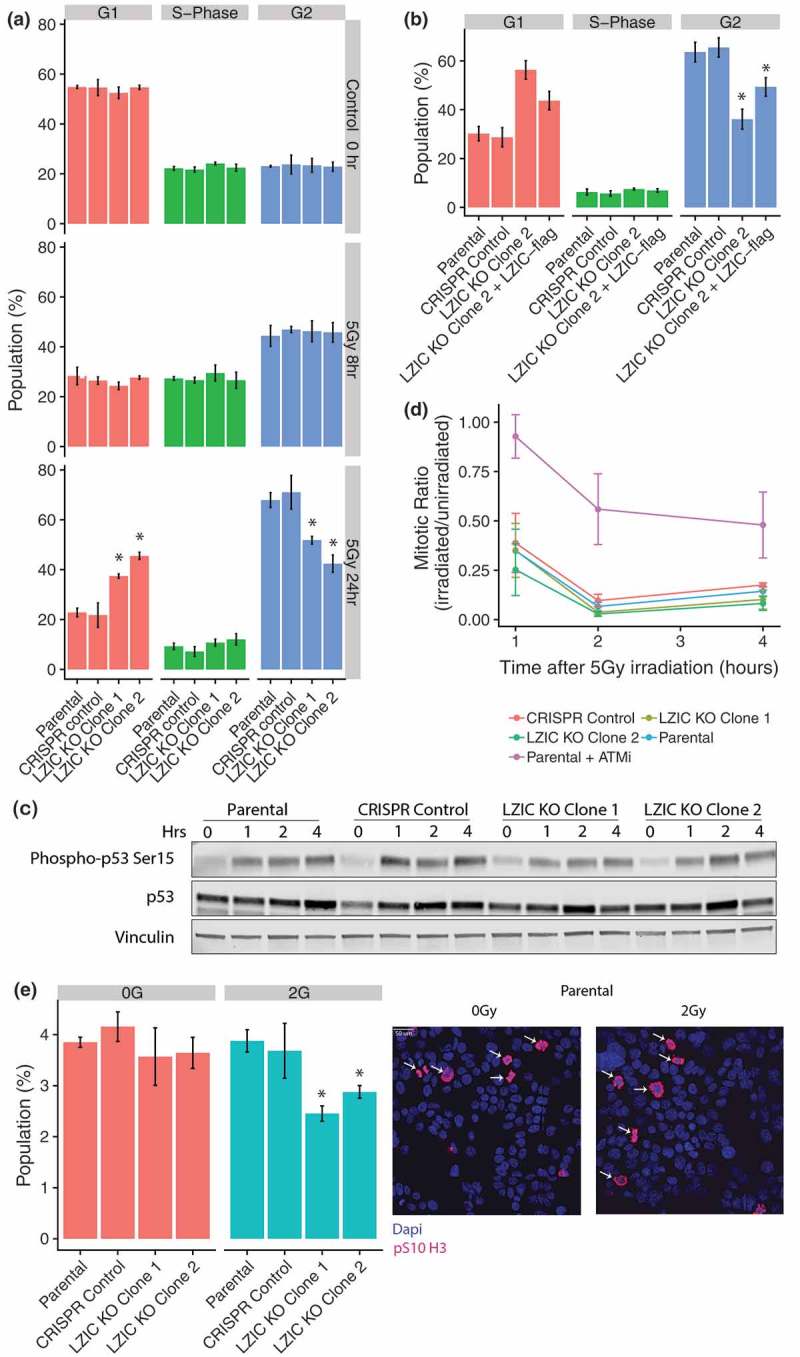
Late G2/M checkpoint arrest is perturbed following LZIC loss. (A) Cell cycle analysis of cell lines by propidium iodide staining. Cell lines were treated with 5 Gy IR and following a 24hr incubation harvested for analysis. Graphs are based on four separate biological repeats (B) Cell cycle analysis of LZIC KO Clone 2 and stable re-expression of LZIC-flag. Graphs based on three separate biological repeats. (C) p53 phosphorylation status in all cell lines, over a 4-h time course, following treatment with IR. A representative blot is shown from three separate biological repeats. (D) Activation of early G2/M checkpoint induction, following treatment with IR. The inclusion of Parental + ATMi provides a positive control for loss of early G2/M checkpoint activation. Graphs based on three separate biological repeats. (E) Quantification of phosphorylated serine 10 on Histone 3. Cells were treated with 2 Gy IR and harvested at 24 h. Images indicate staining profile with arrows to denote the quantified cells. Graphs based on three separate biological repeats. All statistical significance was determined with unpaired student T-Test, * = p-value < 0.05, n.s = non-significant. CRISPR control and LZIC KO Clones were compared to the parental line.

Our transcriptomic analysis found dysregulation of mRNA for critical G2/M checkpoint regulatory genes following treatment with IR in LZIC KO. Altered abundance of cyclin B1 and SFN, in particular, have been linked to progression through the G2/M transition with damaged DNA [26]. Therefore, we used flow cytometry to assess changes in cell cycle distribution in LZIC KO cells following IR treatment. The parental line and an additional LZIC knockout line (LZIC KO Clone 2) were included in this analysis to increase the robustness of derived conclusions (Supplementary Figure 3A & 3B). We observed G2/M checkpoint induction in all cell lines at 8-h post-IR (Figure 2A middle panel). Interestingly, when measured at 24hr post-IR LZIC KO cell lines showed a significantly reduced G2/M population, with a concurrent increase of cells present in the G1 phase (Figure 2A bottom panel). This effect was specific to exposure to IR, since cells treated with camptothecin (CPT), cobalt chloride (CoCl_2_), or Ultra-violet light (UV) showed no phenotype (Supplementary Figure 4A and 4B). To confirm that altered cell cycle distribution was LZIC KO specific and not due to off-target effects, a FLAG-tagged LZIC cDNA was stably introduced into the LZIC KO Clone 2 line. The expression of exogenous LZIC protein was lower than endogenous levels (Supplementary Figure 2A), despite this, exogenous LZIC partially reversed the KO phenotype confirming its specificity (Figure 2B). While the data suggests a defective G2/M checkpoint, the activation of G1 checkpoint following IR treatment was assessed. We used phosphorylation of p53 serine 15 (Ser 15) as a marker of G1 checkpoint signaling induction as it occurs in response to DNA damage and promotes association with p53-responsive promoters [27]. The phosphorylation of p53 Ser 15 is consistent across all the cell lines indicating correct induction of G1 checkpoint signaling irrespective of LZIC loss (Figure 2C).

Two G2/M checkpoints have been characterized: a minor immediate (within 1-h post-IR) ATM-dependent checkpoint and a major ATM-independent G2 accumulation checkpoint [5]. To determine whether induction of early G2/M checkpoint was perturbed, techniques demonstrated by Xu, et.al, were utilized [5]. ATM inhibitor-treated cells were utilized as an experimental control and show an increase in the mitotic ratio relative to the WT cells, indicating loss of the ATM-dependent early checkpoint (Figure 2D). In contrast, the LZIC KO cell lines show no deviation from the WT at the time points measured indicating a correct activation of the early G2/M checkpoint. Finally, the phosphorylation of histone 3 Serine 10 (pS10 H3) occurs upon entry into late G2 and persists until rapid dephosphorylation occurs in early G1 [28]. Quantification of the pS10 H3 population gives a further measure of those cells present in late G2 and mitosis. We found that at 24-h post-IR LZIC KO cells had a reduced positively stained population (Figure 2E). We conclude that LZIC KO cells successfully induce activation of cell cycle checkpoints but fail to sustain the late G2/M checkpoint and proceed to mitosis prematurely.

### Defective signaling downstream of Chk1 in LZIC KO cells

**Figure 3.**
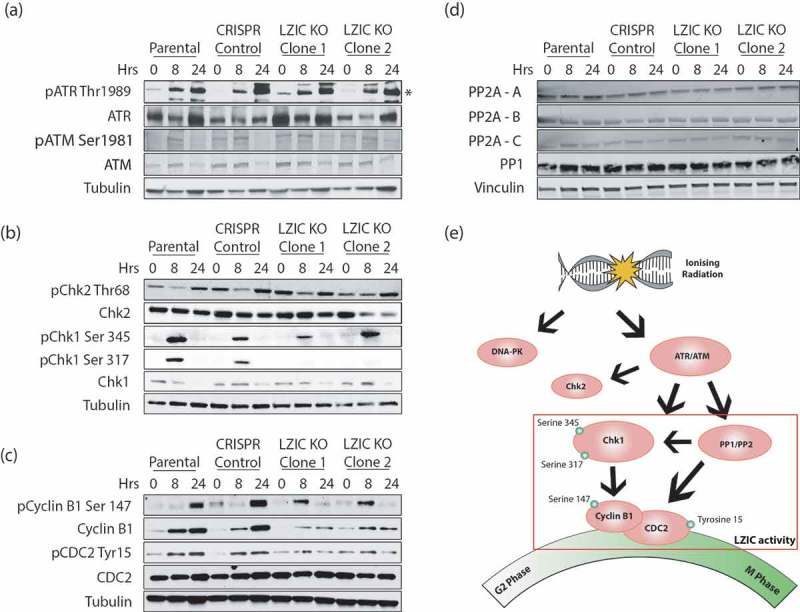
Cellular signaling for activation of G2/M checkpoint in response to IR is perturbed in LZIC KO cells. (A) Analysis of ATR and ATM phosphorylation status at 8 and 24-h post-treatment with 5 Gy IR. (*) indicates protein band corresponding to ATR protein. (B) Western blot analysis of checkpoint proteins at 8 h and 24 h in all cell types following treatment with 5 Gy IR. (C) Western blot analysis of Mitosis promoting factors at 8 and 24 h following treatment with 5 Gy IR. (D) Western blot analysis of major G2/M phosphatases, PP1 and PP2A, at 8 and 24 h following treatment with IR. (E) Schematic diagram of regulatory cascade showing key proteins involved in the G2/M cell cycle progression and their DNA damage-induced phosphorylation sites. All western blots shown are representative image of three separate biological repeats.

The ATR and ATM kinases are essential for the establishment of the G2/M checkpoint following damage induction (Figure 3D) [11]. The activity of ATR and ATM following treatment with IR is regulated by phosphorylation on specific activation residues [29,30]. Analysis of canonical ATR and ATM activation sites show no impact of LZIC loss upon phosphorylation following IR treatment (Figure 3A). The major cell cycle targets of these kinases are both Chk1 and checkpoint protein 2 (Chk2). While Chk1 is the master regulator of G2/M, checkpoint interplay with Chk2 has been observed [31]. Analysis of Chk2 expression levels and activation showed no deviation between LZIC KO cells and control lines. In contrast, the phosphorylation of Chk1 serine 317 was reduced in the absence of LZIC (Figure 3B). The phosphorylation status of Chk1 has a direct impact upon its function, particularly, serine 317 which can reduce the activity of the other two major activation sites serine 296 and serine 345 [12]. Therefore, the phosphorylation status of downstream components reliant on Chk1 activation was analyzed.

The mitosis promoting factor (MPF) is a complex containing cyclin B1 and CDC2 [32]. Phosphorylation of CDC2 at tyrosine 15, a DNA damage-induced phosphorylation site, occurs through a Chk1 mediated pathway and was reduced in LZIC KO clones [33]. Given interdependence between the MPF components, we further investigated the status of cyclin B1 in this condition, as the expression levels of cyclin B1 and phosphorylation of cytoplasmic to nuclear import sites directly effect the progression of cells through mitosis. LZIC KO cells showed reduced expression levels of cyclin B1 at 8-h and 24-h post-IR. Furthermore, phosphorylation of cyclin B1 at serine 147, a site involved in nuclear shuttling, was aberrant in LZIC KOs with peak phosphorylation occurring at 8-h post-IR relative to 24 h seen in control cell lines (Figure 3C) [34]. This data suggests that the MPF complex regulation is altered in response to IR treatment, following LZIC loss, facilitating progression through the G2/M checkpoint into mitosis.

The phosphorylation status of Chk1 is controlled by the interplay between the PIKK proteins and removal of phosphorylation by the protein phosphatase family. To assure that LZIC loss did not lead to loss of phosphatase expression, overall expression of protein phosphatase 1 (PP1) and protein phosphatase 2 A (PP2A) was conducted. We show that the overall expression levels of the phosphatases are unchanged (Figure 3D). We conclude that LZIC operates downstream of PIKK signaling and that LZIC KO cells show a selective defect in the execution of IR-induced signaling which converges on the MPF (Figure 3E).

### Loss of LZIC leads to genome instability and poor prognosis for clear renal cell carcinoma

**Figure 4.**
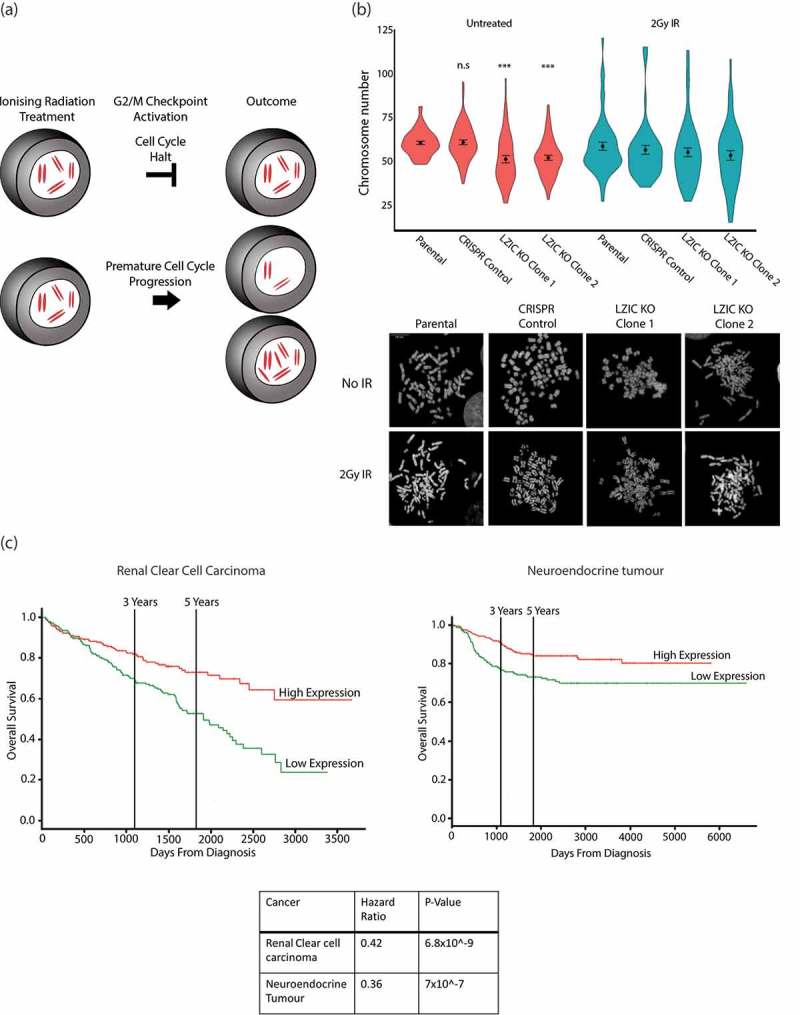
Loss of LZIC leads to genome instability and poor prognosis in clear cell renal carcinoma. (A) Schematic outlining the development of aneuploidy following loss of G2/M checkpoint control. (B) Metaphase spread quantification of chromosome numbers from Parental, CRISPR control line, and LZIC KO Clone 1 and 2. Data from 3 biological repeats counting at least 17 spreads per replicate. Statistical significance was determined using unpaired student T-Test, *** = p-value < 0.001, n.s = non-significant. CRISPR control and LZIC KO clones were compared to the parental line in the untreated condition. (C) Kaplan Meier plot showing overall survival of patients stratified by LZIC expression. The calculated hazard ratios and significance is also included.

A premature release of cells from the G2/M checkpoint increases the chance of chromosome loss and the development of aneuploidy (Figure 4A) [35]. Cells were either left untreated or exposed to IR and metaphase spreads were used to determine chromosome numbers. Under basal conditions, LZIC KO cell lines showed a reduced number of chromosomes when compared to controls. Similar chromosome loss was observed in control cells following IR exposure (Figure 4B). Notably, the genome instability observed in LZIC cells following IR does not increase beyond observed levels in the untreated population. Analysis of cell viability following treatment with IR indicates an increased sensitivity for LZIC KO, which could indicate that the population with increased genome instability are lost (Supplementary Figure 4C). These data suggest that LZIC KO cell lines had spontaneously undergone chromosome loss before IR treatment and that IR-induced instability can generate an equivalent outcome in control cells.

Next, we analyzed available cancer patient databases of RNA-seq data to determine whether there was a correlation of LZIC RNA expression levels with patient prognosis [36]. Although LZIC RNA expression correlated with poor patient survival for a range of cancers, the most striking effect was observed for the clear renal cell carcinoma and neuroendocrine tumors, in which reduction of LZIC expression correlated with a severe decrease in average patient survival times (Figure 4C).

## Discussion

A major treatment modality for cancer is IR, which is used in isolation or in combination with small molecular inhibitors and chemical chemotherapy. The identification of biomarkers for sensitivity to IR is important for improving response rates to this treatment. LZIC expression was shown to be specifically downregulated during the development of IR-initiated oncogenesis [21]. However, the cellular function of LZIC is currently unknown. This investigation aimed to identify the role of LZIC within the cell, and more specifically, the IR response cascade.

This study has generated human LZIC KO cell lines to investigate the effect of LZIC loss on the transcriptomic response to IR. From these data, we can conclude that following IR treatment, LZIC acts to regulate the cell cycle checkpoint cascade, more specifically at the G2/M checkpoint. To our knowledge, this is the first study to suggest such a function for LZIC. In general, the increased activity of WNT signaling proteins at the G2/M checkpoint, and during mitotic spindle assembly, has been widely characterized [37]. One example of which is the interplay between β-catenin and DNA ligase IV being an important radioresistance determinant [38]. Therefore, this finding agrees with current roles for WNT signaling proteins. In addition, WNT signaling is an important pathway during oncogenesis, with the identification of altered LZIC regulation having been established in multiple cancers [19–21]. The hypothesis presented here suggests reduced LZIC expression is linked to induced oncogenesis by decreased checkpoint control.

The transcriptomic analysis of LZIC KO cells identified altered MYC signaling in untreated and treated conditions. This suggested that the altered regulation of this pathway is not IR specific and is, instead, a direct response to the loss of LZIC. The regulation of MYC signaling by WNT pathway proteins, for example, the upregulation of c-myc by β-catenin, can promote cell proliferation and differentiation [39,40]. Further investigation would be required to determine a role for LZIC in the regulation of the MYC pathway. However, these data suggest a similar role to canonical WNT signaling components.

The analysis of pS10 H3 levels in LZIC KO cells following IR identified a reduced number of cells in late G2 and mitosis compared to control lines. Previous literature shows that following release from the G2/M checkpoint, the mitotic population significantly increases [5]. However, the release from the G2/M checkpoint can begin as early as 12-h post-IR [5]. In this case, we hypothesize that LZIC KO cells undergo early G2/M checkpoint release prior to the 24-h time point, which causes the majority of the population to have passed through mitosis into G1. In G1 the mitotic pS10 H3 is rapidly lost, decreasing the observable population. These data are supported by the cell cycle analysis at 24 h indicating an increased G1 population (Figure 2A).

The phosphorylation of Chk1 S317 is mediated by ATR. The loss of this phosphorylation event has been shown to perturb the function of surrounding phosphorylation sites S345 and S296 [12]. Therefore, the reduced phosphorylation of this site in LZIC KO cells could have a detrimental impact on Chk1 activity. Interestingly, the altered phosphorylation status of the MPF components is downstream of both Chk1 and the protein phosphatase family [41]. We hypothesize that while expression levels of PP1 and PP2 are not altered, it is the interplay between these proteins and Chk1 which leads to the defect of checkpoint control.

The genome instability observed in LZIC KO cells is significant under basal conditions. The link between a dysfunctional G2/M checkpoint and increased genome instability has been previously shown [35]. In addition, a damage threshold must be overcome to successfully activate the spindle assembly checkpoint [42]. LZIC KO cells do not show changes to cell cycle prior to damage with IR; however, it is possible that LZIC cells possess a defect which increases the number of cells progressing through cell cycle with damage. We stipulate that LZIC cells reduce the fidelity of the G2/M checkpoint, which over time will yield the phenotypes of genome instability.

Following LZIC KO the transcriptional signatures identified showed altered regulation of multiple genes with known functions in neuronal differentiation and development. A previous study has identified LZIC as a factor required for the correct development of the zebrafish brain midline [18]. The high conservation of LZIC in zebrafish and the interaction with genes associated with the development may provide a basis for further investigation into the regulation of these pathways, leading to a mechanism by which this process is controlled.

Collectively our data classifies LZIC as functionally involved in the IR response cascade. Clearly more mechanistic data on LZIC protein and its interacting factors are necessary to fully comprehend the contribution of this protein to mammalian DDR. However, even at this early stage, our data are suggestive of the usefulness of LZIC as a biomarker for patient stratification, given that its expression is strongly correlated with survival of patients suffering from clear cell renal carcinoma.

## Materials and methods

### LZIC protein evolutionary conservation analysis

National center for bioinformatics information (NCBI) nucleotide sequence database was interrogated manually and the nucleotide sequences for Human, Mouse, Xenopus, Zebrafish, Nematodes, and Slimemold were acquired. The previously identified domains were aligned, by ClustalW [43], and a percentage conservation score calculated by assessing the number of nucleotides conserved between sequences by the equation – total number of conserved nucleotides (Analyzed species)/total number of nucleotides (Humans).

### Cell culture

HEK293 were cultured at 5% CO_2_ in Dulbecco’s-modified Eagle’s medium (DMEM), supplemented with 4.5 g/l D-glucose, GlutaMAX (Life Technologies, Carlsbad, CA, USA) and 10% fetal bovine serum.

### LZIC knock-out line generation

LZIC-targeting CRISPR-based knockout plasmid kit was purchased from Origene. HEK293 cells were transfected with plasmids provided in Origene kit using Lipofectamine LTX. Cells were cultured for eight passages before addition of antibiotic selection, as per manufacturer’s instructions. Cells were reseeded and treated with Puromycin (0.5μg/ml) and individual colonies were selected, by the use of cloning discs. Individual colonies were expanded and screened for LZIC expression by western blot. LZIC-Flag CDS was reintroduced into LZIC knockout (KO) Clone 2 by Lentiviral transduction. Prior to transduction LZIC KO clone 2 was transfected with Cre recombinase plasmid to remove puromycin resistance cassette from a cell line.

### Microarray analysis of LZIC KO cells

All clones were plated in duplicate for both untreated and IR treated conditions. After 24 h, cells were exposed to 5 Gy IR and incubated for a further 24 h before harvesting. Untreated cells were harvested 48-h post seeding. Cells were harvested using trypsin and EDTA before RNA extraction using RNeasy kit (Qiagen) as per manufacturers instructions. Samples were subsequently labeled by low input quick amp labeling (Agilent Technologies) as per manufacturer’s instructions in one-colour microarray-based gene expression analysis. The chipset reference was G4858A, GE 8 x 60K with design 039494 V3. 100 ng of RNA was used for analysis. Microarray was imaged on DNA microarray scanner with Surescan high-resolution imaging (Agilent technologies). The resulting raw data were analyzed using the R package Limma as conducted in previous studies [44,45]. Gene set enrichment analysis (GSEA) was conducted by comparing gene sets to the Molecular Signatures Database (MSigDB) [24].

### qPCR analysis

HEK293 cells and LZIC KO clones were grown for 24 h prior to treatment with 5 Gy IR. Cells were harvested by trypsinization prior to extraction of RNA using RNeasy kit (Qiagen kit). 1000 ng of extracted RNA was reverse transcribed to cDNA by Superscript II (Thermo Fisher Scientific) as per the manufacturer’s instructions. The qPCR was conducted using SYBR green reagent (Applied Biosystems, Thermofisher) and plates were analyzed on Quantstudio 6 flex (Applied Biotechnologies). Delta-delta ct calculation was conducted using GAPDH as a reference gene. Primers sequences used are shown in Table 1.

**Table 1.** Primer sequences for qPCR.

Gene Name	Forward Primer (5’-3’)	Reverse Primer (5’-3’)
GapDH	GGAGTCAACGGATTTGGTCGTA	GAATTTGCCATGGGTGGAAT
LZIC	AGTCTCTACAGACCTTGGCTC	ACAAGCTTCTGCACCATGTC
CCBN1	AACTTTCGCCTGAGCCTATTTT	TTGGTCTGACTGCTTGCTCTT
SOX11	CGGTCAAGTGCGTGTTTCTG	CACTTTGGCGACGTTGTAGC
NREP	CTGTCTTTCTAGCATGTTGCCC	CCAGGGAGACCAACAGACAA
FLNA	GTCACAGTGTCAATCGGAGGT	TGCACGTCACTTTGCCTTTG
POU3F2	TTGTGTTGCCCCTTCTTCGT	TTGCCTTCGATAAAGCGGGT
CPNE7	CACCCTGGGGCAGATTGTG	TCACCGTGATGGTGGACTTG
SFN	CGCTGTTCTTGCTCCAAAGG	ATGACCAGTGGTTAGGTGCG
LGALS3	GGGCCACTGATTGTGCCTTA	TCACCGTGCCCAGAATTGTT
IFI30	TACGGAAACGCACAGGAACA	CAGGCCTCCACCTTGTTGAA

### Western blotting

HEK293 and CRISPR lines were seeded and 24 h following either left untreated or exposed to 5Gy IR. The cells were then harvested at the stated time points following IR. Cells extracts were generated by the addition of RIPA buffer (150mM NaCl, 50nM Tris pH 7.5, 1% Triton X-100, 0.5% sodium deoxycholate, 0.1% SDS, 1mM DTT, 0,4mM PMSF, Protease inhibitor cocktail) and sonication for 2 × 10 s. Samples were loaded with 1x Laemmli buffer before being heated to 90°C for 10 min. Samples were run on SDS-Page gels and transferred to nitrocellulose membrane by use of Bio-Rad Transblot Turbo. The antibodies used were: LZIC (Bethyl, 1/1000), Tubulin (Sigma Aldrich, 1/5000), pChk2 Thr68 (Cell signalling, 1/2000), Chk2 total (Bethyl, 1/2000), pChk1 S345 (Bethyl, 1/2000), pChk1 S317 (Bethyl, 1/2000), Chk1 total (Bethyl, 1/2000), pATR Tyr1981 (Cell signalling, 1/1000), ATR total (Cell signalling, 1/1000), pCyclin B1 Ser147 (Cell signalling, 1/2000), Cyclin B1 (Cell signalling, 1/2000), pCDC2 Tyr15 (Cell signalling, 1/2000), and CDC2 total (Cell signalling, 1/2000). Secondary goat antibody was horseradish peroxidase conjugated with reactivity against mouse or rabbit (Thermo Fischer Scientific). All further antibodies were analyzed using LICOR system using goat-secondary with conjugated fluorescence. PP2A – subunit A (Cell signalling, 1/1000), PP2A – subunit B (Cell signalling, 1/1000), PP2A – subunit C (Cell signalling, 1/1000), PP1 (Santa Cruz, 1/1000), pATM Serine 1981 (Cell signalling, 1/1000), ATM total (Cell signalling, 1/1000), Vinculin (Abcam, 1/5000), p53 total (Santa Cruz, 1/1000), p-p53 Serine 15 (Cell signalling, 1/1000).

### Cell cycle analysis

#### 

Twenty-four hours after seeding HEK293 cells and CRISPR clones were treated with IR (5 Gy), camptothecin (20 μM), cobalt chloride (200 μM), or UV (20 mJ) and incubated 24 h. Cells were incubated for further 8 and 24 h and then harvested. After washing with PBS, ice-cold 70% ethanol was slowly added under slight agitation. Cells were left at 4°C for 24 h to fix, PBS washed, and Propidium Iodide and RNase A were added to final concentration of 10ug/ml and 100ug/ml, respectively. Samples were heated to 37°C for 30 min and then incubated at 4°C for at least 4 h before reading. Flow cytometry analysis of cells was conducted on a BD biosciences FACS canto.

#### Early G2/M checkpoint activation

This method was conducted as shown in Xu, et.al. 2002 [5]. One set of control cells were additionally treated with ATMi (10μM final concentration, Sigma Aldrich) 1 h prior to exposure to 5 Gy IR. Cells were stained with pS10 H3 antibody (Cell Signalling, 1/100) and incubated with Goat-anti-rabbit 488 (Abcam, 1/500). The cells were then analyzed on Attune NXT (Life Technologies).

#### Immunofluorescence

Parental HEK293 and CRISPR clones were seeded and treated with 2 Gy IR. The cells were incubated for 24 h before supernatant was removed and cells washed with PBS. Four percent Paraformaldehyde was used to fix cells for 10 min at room temperature before treatment with blocking buffer (0.3% triton X-100 in PBS supplemented with 5% goat serum). Fixed cells were treated with primary antibody overnight at 4°c. Cells were washed 3x with PBS before the addition of secondary antibody and incubated at room temperature for 1 h. Cells were mounted with hard set mounting medium (vector hard set mounting medium, Vector labs). Images were acquired using a Zeiss LSM 510 and images were processed with ZEN 2009 software. Primary antibody – Phospho-serine 10 Histone 3 antibody (Cell Signalling Technology, 1/1000). Secondary antibody was goat anti-rabbit with conjugated Cy5 (Thermo Fischer Scientific).

#### Metaphase spread analysis

Parental HEK293 and CRISPR clones were seeded and treated with 2 Gy IR before incubating for 48 h. Cells were harvested by trypsinization and centrifuged at 300 g 5 min before swelling buffer (75 mM KCl) was added. The cell pellet was incubated for 10 min at room temperature before addition of a fixative solution (Methanol and acetic acid 3:1 ratio). The Cells were centrifuged at 200 g for 5 min and the supernatant was removed. This step was repeated twice. Pellet was suspended in fixative to give cell suspension and dropped from a height of 30 cm onto slides (Superfrost plus, Thermo scientific). Slides were dried at room temperature for 2 min before steaming for 10 s. Slides were left in a humidity box overnight to dry. Cells were stained with Dapi (1/5000) diluted in PBS and then mounted. Images were acquired using a Zeiss LSM 510. Manual counts were conducted of spreads to determine chromosomal numbers.

#### Cell viability assay

Cells were treated with 0, 40, 60, or 80 Gy IR before a 24-h incubation. The WST-1 reagent (Sigma Aldrich) was used and data analyzed as per manufacturers instructions. With the following deviations, the WST-1 reagent was added 2 h prior to absorbance quantification. With the absorbance being read by Powerwave XS2 plate reader (BioTek).

#### Kaplan-meier plot generation

The PROGgene V2 database was used to generate Kaplan-Meier plots for LZIC expression in cancers [36]. The overall survival of patients was analyzed with no stratification apart from LZIC expression.
