# Supplementary material for: Leucine zipper and ICAT domain containing (LZIC) protein regulates cell cycle transitions in response to ionizing radiation
Source: Cell Cycle. 2019 Apr 19;18(9):963–75. doi: 10.1080/15384101.2019.1601476 (PMC6527300; doi:10.1080/15384101.2019.1601476)

Supplementary Figure 1

(A)

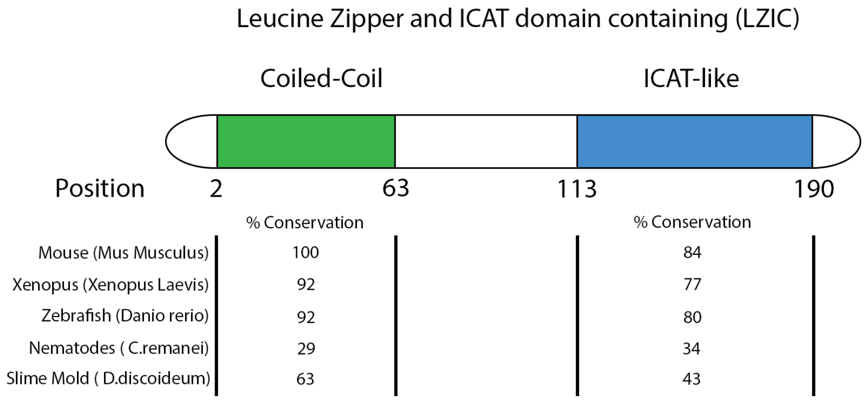

(B)

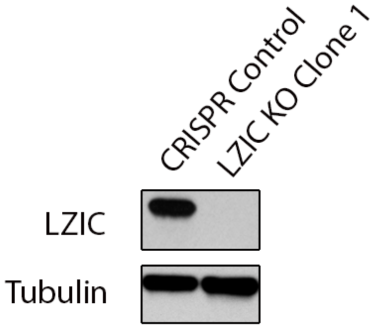

(C)

CRISPR Control - Untreated vs 5Gy IR

Gene	Log Fold Change	Adjusted p-value
PPP1R15A	1.067	0.020
lnc-C16orf13-3	1.024	0.027
ACLY	0.932	0.071
RLTPR	0.921	0.038
RNF122	0.903	0.023
COX2	0.874	0.100
IRX4	0.843	0.094
GFRA2	0.818	0.056
RNF44	0.806	0.070
ENST00000604257	0.801	0.070

Gene	Log Fold Change	Adjusted p-value
A_33_P3234540	-1.498	0.071
lnc-RAB2A-1	-1.167	0.086
HIST1H2AC	-1.048	0.045
LOC102723989	-1.023	0.032
PPA2	-0.999	0.072
lnc-APBA1-2	-0.985	0.038
HIST3H2BB	-0.968	0.063
ENST00000511579	-0.968	0.053
HIST1H2AI	-0.940	0.025
lnc-VAX1-1	-0.900	0.056

LZIC KO Clone 1 - Untreated vs 5Gy IR

Gene	Log Fold Change	Adjusted p-value
CDC42EP1	1.241	0.056
GAS5	1.194	0.005
A_32_P230825	1.130	0.006
CCNB1IP1	1.106	0.006
C6orf48	1.085	0.014
TMEM200C	1.061	0.068
lnc-GNA12-2	1.032	0.063
GADD45A	1.016	0.056
ENST00000511103	1.001	0.021
PSPH	0.999	0.032

Gene	Log Fold Change	Adjusted p-value
ZBED3-AS1	-1.003	0.032
C3AR1	-0.906	0.056
HSPA1A	-0.882	0.058
MT2A	-0.869	0.068
HIST1H2BO	-0.816	0.076
MT1L	-0.776	0.078
lnc-RTL1-2	-0.757	0.092
AKAP5	-0.757	0.063

Untreated - CRISPR Control vs LZIC KO Clone 1

Gene	Log Fold Change	Adjusted p-value
lnc-TEFM-2	1.142	0.055
MIATNB	1.103	0.055
LOC102725381	1.046	0.031
TLR5	1.012	0.049
MEG3	1.005	0.040
TESC	0.948	0.049
ENTPD1	0.942	0.045
RAD51-AS1	0.938	0.055
PRPH	0.913	0.040
LXN	0.903	0.076

Gene	Log Fold Change	Adjusted p-value
lnc-PDZD7-1	-1.352	0.065
LOC102725059	-1.280	0.076
lnc-PLXND1-1	-1.045	0.036
HMGB2	-0.954	0.097
FOXQ1	-0.952	0.045
ANXA2R	-0.939	0.080
RPL32	-0.930	0.035
NDUFS4	-0.903	0.031
SNORA67	-0.893	0.045
RPL34	-0.853	0.060

5Gy IR - CRISPR Control vs LZIC KO Clone 1

Gene	Log Fold Change	Adjusted p-value
IDNK	1.111	0.048
ZNF503-AS2	0.960	0.093
MAL2	0.915	0.054
BAIAP2L2	0.874	0.052
CDA	0.859	0.092
BMP2	0.834	0.086
LOC102724532	0.833	0.059
KCNG1	0.820	0.086
LYRM9	0.809	0.078
HMGCL	0.799	0.079

Gene	Log Fold Change	Adjusted p-value
FLNA	-1.089	0.187
CD24	-1.006	0.282
PLK2	-0.958	0.112
MAP3K1	-0.936	0.121
TMA16	-0.865	0.112
CXCR4	-0.862	0.193
CCNB1	-0.844	0.159
TERC	-0.824	0.112
IGFBP7	-0.811	0.139
PDLIM3	-0.787	0.139

Supplementary Figure 2

(A)

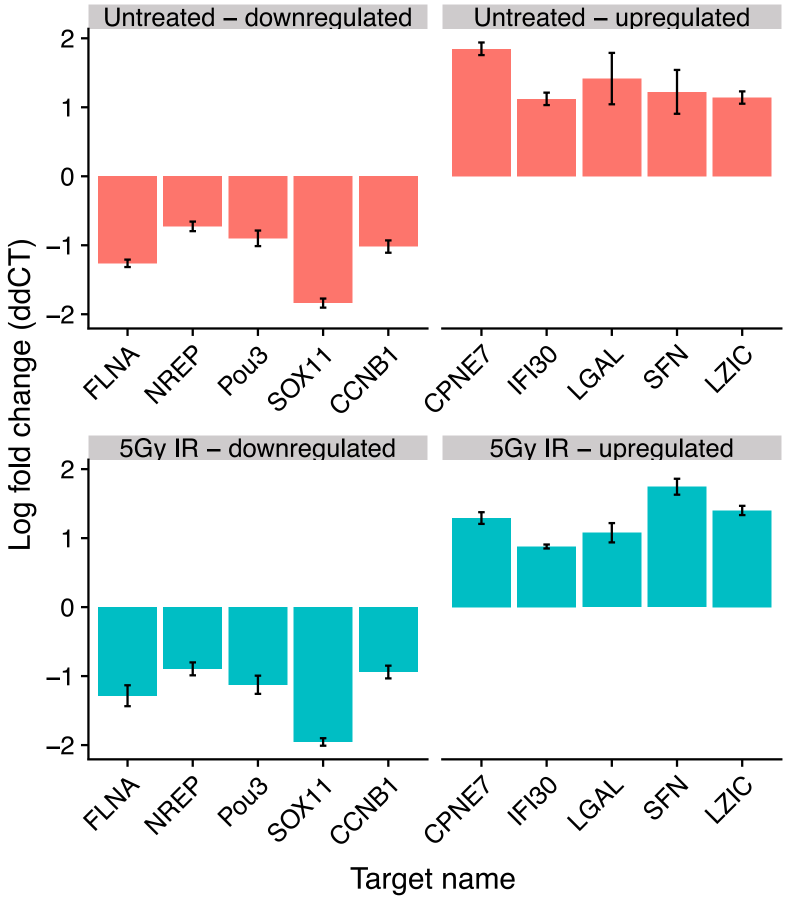

(B)

Gene	Log Fold Change - 0hr	Log Fold Change – 24hr
CPNE7	1.719	1.778
IFI30	1.584	1.431
LGALS3	1.543	1.57
SFN	1.359	1.695
LZIC	0.904	1.428
POU3F2	-0.892	-1.054
FLNA	-0.625	-0.089
CCNB1	-0.874	-1.216
NREP	-1.12	-1.212
SOX11	-1.806	-1.409

(C)

Hallmark Gene Group	FDR q-val
MYC Targets	0.027
E2F Targets	0.067
Notch Signaling	0.111
WNT/Beta Catenin Signaling	0.112
TGF Beta Signaling	0.092
G2/M Checkpoint	0.153
IL2/STAT5 Signaling	0.195
Unfolded Protein Response	0.201
DNA Repair	0.195
Mitotic Spindle	0.227

Supplementary Figure 3

(A)

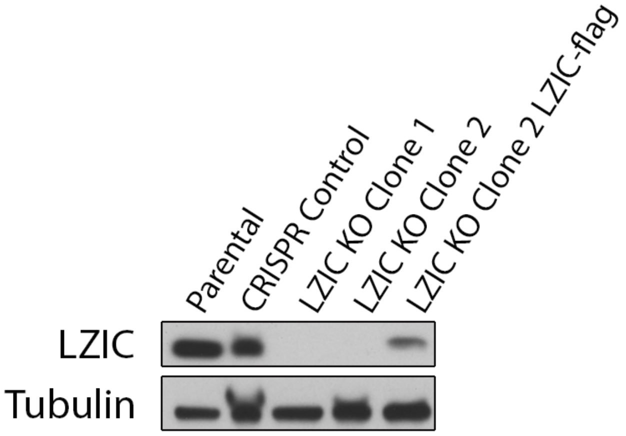

(B)

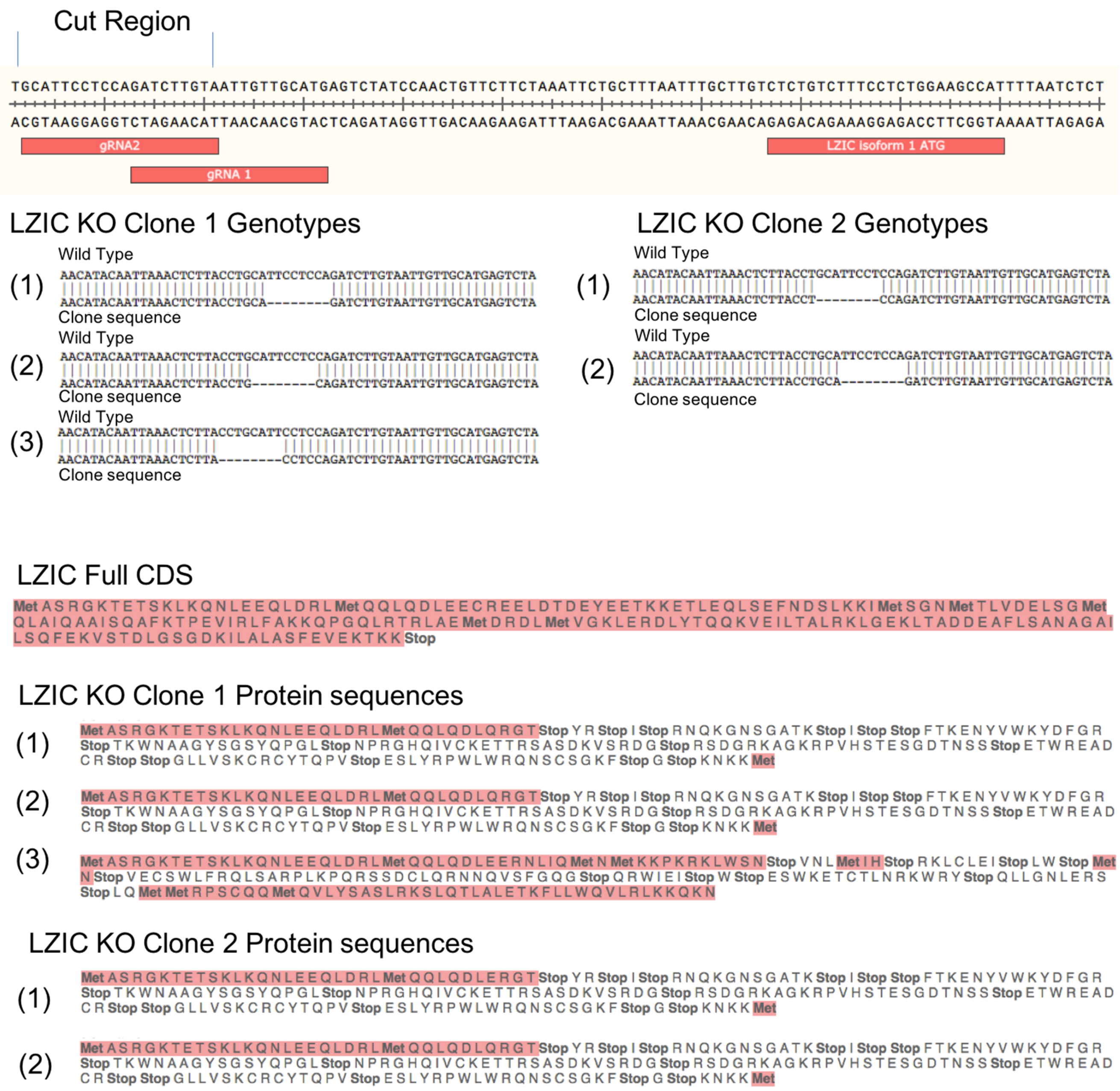

Supplementary Figure 4

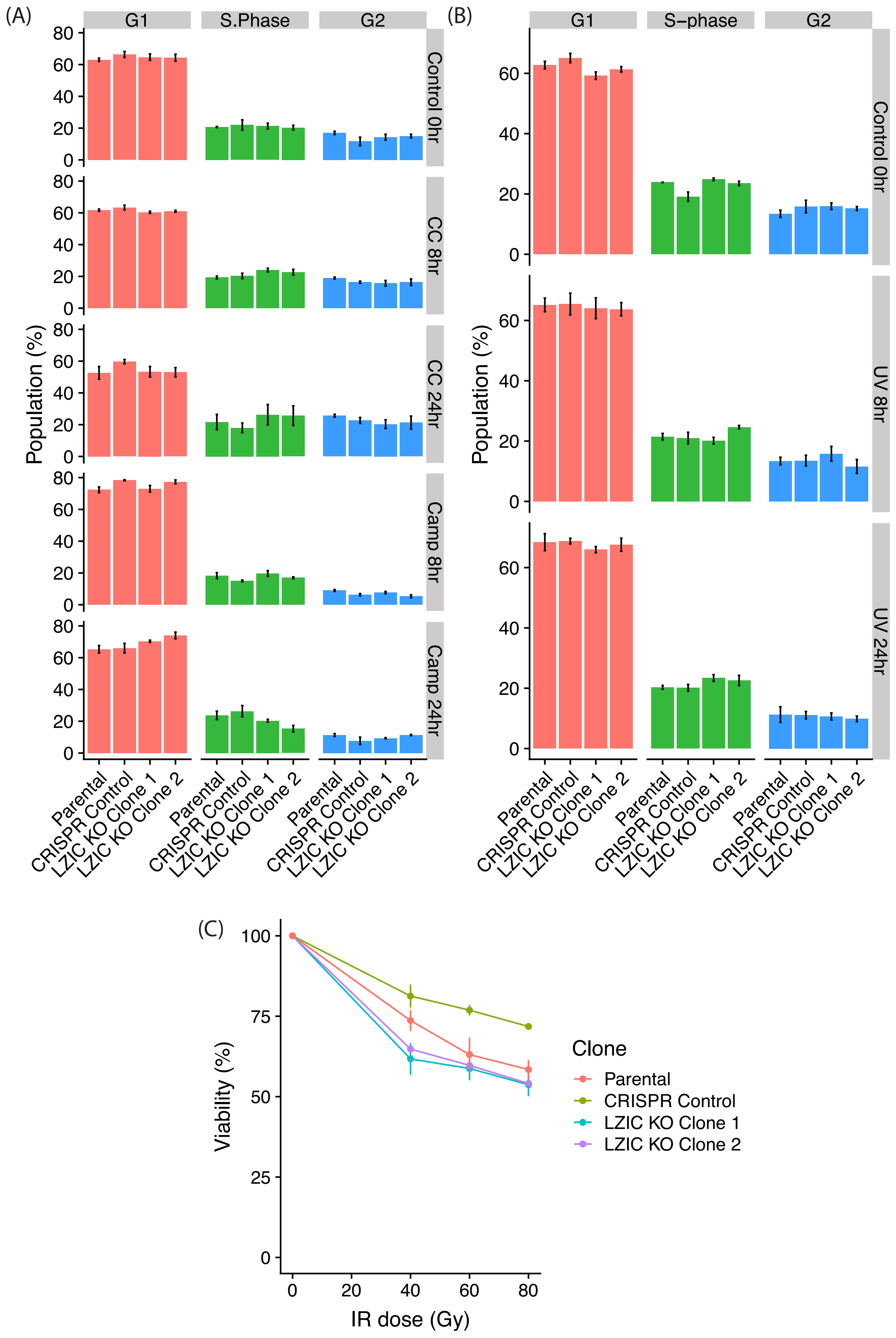

Supplement: Supplemental Material [file kccy-18-09-1601476-s001.zip › Supplementary information/Pages from Supplementary.pdf]
