# Supplementary material for: Leucine zipper and ICAT domain containing (LZIC) protein regulates cell cycle transitions in response to ionizing radiation
Source: Cell Cycle. 2019 Apr 19;18(9):963–75. doi: 10.1080/15384101.2019.1601476 (PMC6527300; doi:10.1080/15384101.2019.1601476)
Supplement: Supplemental Material [file kccy-18-09-1601476-s001.zip › Supplementary information/Supplementary Figures.docx]

Supplementary Figure 1. (A) Schematic representing LZIC protein structure and evolutionary conservation of the two predicted domains. (B) LZIC expression levels following CRISPR mediated KO from HEK293 cells. (C) 10 most highly up and down-regulated genes following log-fold change analysis for all conditions. Only unique genes to each group are included.

Supplementary Figure 2. (A) qPCR validation of gene expression changed observed in microarray data. Based on 3 separate biological repeats. (B) Log-fold changes of gene abundance from microarray analysis for targets validated by qPCR. (C) GSEA of LZIC KO Clone 1 differential gene expression in basal condition.

Supplementary Figure 3. (A) LZIC expression comparison between parental, CRISPR control line, LZIC KO clone 1 and 2, and LZIC KO Clone 2 with flag reintroduction. (B) LZIC KO clone break site sequencing and analysis of resulting protein

Supplementary Figure 4. (A) Cell cycle analysis of Parental, CRISPR control, and LZIC KO Clone 1 and 2 at 24hrs following treatment with camptothecin (Camp) (20 μM) and cobalt chloride (CC) (200μM). (B) Cell cycle analysis of Parental, CRISPR control, and LZIC KO Clones 24hrs following treatment with ultraviolet light (20 mJ). All Graphs based on 3 separate biological repeats. (B) Cell viability analysis of cell lines at 24hrs following treatment with increasing doses of IR. Viability of cells is represented as a percentage of signal shown at 0Gy and the data is representative of 3 separate biological replicates.
